# Methylated TRF2 associates with the nuclear matrix and serves as a potential biomarker for cellular senescence

**DOI:** 10.18632/aging.100650

**Published:** 2014-04-05

**Authors:** Taylor R. H Mitchell, Xu-Dong Zhu

**Affiliations:** Department of Biology, McMaster University, Hamilton, Ontario, Canada L8S 4K1

**Keywords:** TRF2, arginine methylation, nuclear matrix, cellular senescence

## Abstract

Methylation of N-terminal arginines of the shelterin component TRF2 is important for cellular proliferation. While TRF2 is found at telomeres, where it plays an essential role in maintaining telomere integrity, little is known about the cellular localization of methylated TRF2. Here we report that the majority of methylated TRF2 is resistant to extraction by high salt buffer and DNase I treatment, indicating that methylated TRF2 is tightly associated with the nuclear matrix. We show that methylated TRF2 drastically alters its nuclear staining as normal human primary fibroblast cells approach and enter replicative senescence. This altered nuclear staining, which is found to be overwhelmingly associated with misshapen nuclei and abnormal nuclear matrix folds, can be suppressed by hTERT and it is barely detectable in transformed and cancer cell lines. We find that dysfunctional telomeres and DNA damage, both of which are potent inducers of cellular senescence, promote the altered nuclear staining of methylated TRF2, which is dependent upon the ATM-mediated DNA damage response. Collectively, these results suggest that the altered nuclear staining of methylated TRF2 may represent ATM-mediated nuclear structural alteration associated with cellular senescence. Our data further imply that methylated TRF2 can serve as a potential biomarker for cellular senescence.

## INTRODUCTION

Cellular senescence refers to a state of permanent arrest of cell proliferation and it is generally thought to be a response to potentially oncogenic stimuli [1, 2], which include telomere shortening, DNA damage either at telomeres or elsewhere in the genome, strong mitogenic stimuli and epigenomic perturbations. Since it was first described approximately fifty years ago [3], cellular senescence has emerged as an important mechanism linked to both tumor suppression and aging. In most human somatic cells, telomeres shorten with each round of DNA replication, in part because DNA polymerases are unable to fill in the gap generated from removal of the last RNA primer [4]. When telomeres become critically short, they activate a DNA damage response and trigger the induction of cellular senescence [5]. Telomerase, a reverse transcriptase that can replenish the repetitive telomeric DNA *de novo*, is able to circumvent telomere shortening and allows cells to gain unlimited growth potential, a feature associated with cancer. Therefore cellular senescence is widely considered as a tumor suppressive mechanism. In addition, cellular senescence is also implicated in tissue repair and inflammation associated with aging and cancer progression [1, 2].

The nuclear matrix is a filamentous network of protein, DNA and RNA that is refractory to high salt extraction [6-8]. This structure serves as an architectural skeleton to the nucleus and provides support for chromatin organization and various nuclear functions including DNA replication, transcription and DNA repair [8]. Changes in the composition of the nuclear matrix have been observed in senescent cells [9]. Alterations in the nuclear matrix have been implicated in restricting cellular proliferation in mortal human fibroblasts [10]. Furthermore, nuclei with abnormal nuclear structure are found to accumulate in aged and prematurely senescent cells [11, 12].

Telomeres, heterochromatic structures found at the ends of linear eukaryotic chromosomes, function to protect natural chromosome ends from being recognized as damaged DNA. Mammalian telomeric DNA consists of tandem repeats of TTAGGG and is bound by a six-subunit protein complex [13, 14], referred to as shelterin or telosome, which is composed of TRF1, TRF2, TIN2, POT1, TPP1 and hRap1. Telomeres are attached to the nuclear matrix [15, 16] and components of the shelterin complex have been reported to be associated with the nuclear envelope as well as the nuclear matrix [10, 17-19]. TIN2L, an isoform of TIN2, has been suggested to mediate the interaction between telomeres and the nuclear matrix [17] whereas hRap1 has been implicated in tethering telomeres to the nuclear envelope [18]. TRF2, a shelterin protein that interacts directly with both TIN2 and hRap1 [20-23], has also been implicated in mediating the interaction between telomeres and the nuclear matrix [19]. However, little is known about the role of post-translational modification in mediating the interaction between shelterin proteins and the nuclear matrix.

TRF2, a subunit of the shelterin complex, binds to duplex telomeric DNA [24, 25] and plays a crucial role in telomere protection [26]. TRF2 contains an N-terminal basic domain rich in glycines and arginines (the GAR domain), a central TRFH dimerization domain, a flexible linker region and a C-terminal Myb-like DNA binding domain [24, 25]. It has been shown that loss of TRF2 from telomeres through either TRF2 knockout or overexpression of a dominant-negative allele of TRF2 promotes the formation of telomere end-to-end fusions [26, 27]. On the other hand, overexpression of TRF2 lacking the basic/GAR domain induces telomere rapid deletion [28] whereas overexpression of TRF2 carrying amino acid substitutions of arginines to lysines in the basic/GAR domain promotes the formation of fragile telomeres [29]. In all aforementioned cases, the formation of dysfunctional telomeres resulting from disruption of TRF2 function results in the induction of cellular senescence [26-29].

TRF2 undergoes extensive post-translational modification [30], which in turn regulates its stability, DNA binding and cellular localization. Ubiquitylation of TRF2 by Siah1, an E3 ligase, promotes TRF2 degradation and replicative senescence of human primary fibroblasts [31]. Acetylation, SUMOlyation and poly(ADP-ribosyl)ation have been implicated in modulating TRF2 binding to telomeric DNA [32-35]. TRF2 is phosphorylated in response to DNA damage and this phosphorylation has been implicated in DNA double strand break repair [36, 37]. Arginines in the N-terminal basic/GAR domain of TRF2 are methylated by protein arginine methyltransferase 1 (PRMT1) [29]. Loss of arginine methylation in TRF2 induces DNA-damage response foci at telomeres and triggers cellular senescence [29]. Arginine methylation is also implicated in negatively regulating the amount of telomere-bound TRF2 [29], raising a question as to whether methylated TRF2 is associated with telomeres *in vivo*.

Here we report that the majority of methylated TRF2 is not released by the treatment with high salt buffer and DNase I digestion and that it co-fractionates with lamin A, a component of the nuclear matrix [38, 39], suggesting that methylated TRF2 interacts with the nuclear matrix. We find that methylated TRF2 is largely not localized at telomeres, indicating that association of TRF2 with the nuclear matrix is likely independent of telomeres. We demonstrate that methylated TRF2 dramatically changes its nuclear staining as normal human primary fibroblast cells approach and enter replicative senescence. We find that the altered nuclear staining of methylated TRF2 is predominantly associated with misshapen nuclei and abnormal nuclear matrix folds. Introduction of hTERT into human primary fibroblast cells suppresses the altered nuclear staining of methylated TRF2, suggesting that progressive telomere shortening may contribute to the altered staining of methylated TRF2 in normal primary fibroblasts. In addition to telomere shortening, dysfunctional telomeres and ionizing radiation-induced DNA damage, both of which are potent inducers of cellular senescence, also promote the altered nuclear staining of methylated TRF2. Furthermore, we show that the lack or inhibition of ATM (ataxia telangiectasia mutated), a master regulator of the DNA damage reponse [40, 41], blocks the formation of ionizing irradiation (IR)-induced altered nuclear staining of methylated TRF2, indicating that the formation of the altered nuclear staining of methylated TRF2 is mediated by the ATM-dependent DNA damage response. Taken together, our results reveal that methylated TRF2-associated nuclear matrix undergoes an ATM-mediated structural alteration during the process of cellular senescence. Our data further imply that methylated TRF2 may serve as a potential biomarker for cellular senescence.

## RESULTS

### Methylated TRF2 is associated with nuclear matrix

We have previously reported that PRMT1 methylates arginines in the N-terminal GAR domain of TRF2 and that this arginine methylation negatively regulates TRF2 association with telomere chromatin [29], suggesting that methylated TRF2 is not associated with telomeres *in vivo*. To investigate the nuclear compartmentalization of methylated TRF2, we first subjected hTERT-immortalized BJ (hTERT-BJ) cells to analysis of sequential extraction of the nuclear matrix, which began with the treatment of cells with the CSK buffer to remove the majority of soluble proteins. The treatment of the RSB-magik buffer further removed the cytoskeleton, leaving behind the nuclei and their attached filaments. Digestion of the nuclei with DNase I released chromatin bound proteins and the DNase I-resistant pellet was then further fractionated to release the outer nuclear matrix components by 2M NaCl. The treatment with RNase A disassembled ribonucleo-proteins and the final insoluble pellet contained the core nucleofilament proteins. The DNaseI-resistant fractions including the pellet are referred to as the nuclear matrix-associated fractions.

**Figure 1:**
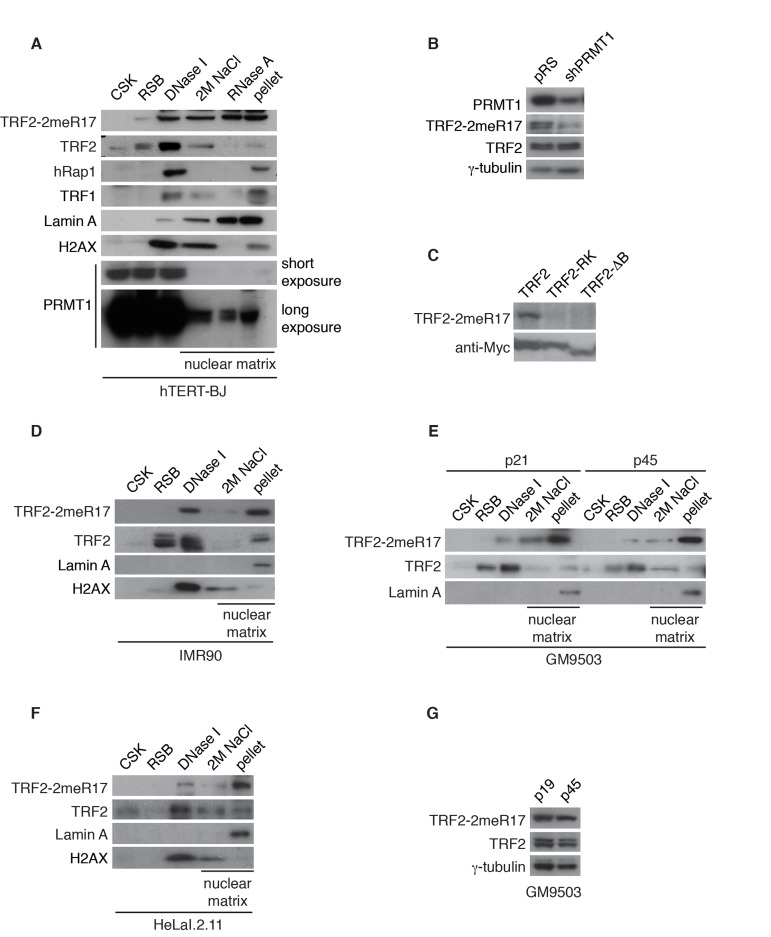
Methylated TRF2 is associated with the nuclear matrix. (**A**) Sequential extraction of the nuclear matrix from hTERT-BJ cells. Immunoblotting was performed with anti-TRF2-2meR17, anti-TRF2, anti-hRap1, anti-TRF1, anti-Lamin A, anti-H2AX, or anti-PRMT1 antibody. (**B**) Western analysis of 293T cells expressing shPRMT1 or the vector alone. Immunoblotting was performed with anti-PRMT1, anti-TRF2-2meR17 or anti-TRF2 antibody. The γ-tubulin blot was used a loading control. (**C**) Western analysis of 293T cells overexpressing Myc-tagged wild type TRF2, TRF2 carrying amino acid substitutions of arginines to lysines (TRF2-RK) or TRF2 lacking the N-terminal GAR/basic domain (TRF2-ΔB). Immunoblotting was carried out with anti-TRF2-2meR17 or anti-Myc antibody. (**D**) Sequential extraction of the nuclear matrix from IMR90 cells. Immunoblotting was performed with anti-TRF2-2meR17, anti-TRF2, anti-Lamin A or anti-H2AX antibody. (**E**) Sequential extraction of the nuclear matrix from GM9503 cells. Immunoblotting was performed with anti-TRF2-2meR17, anti-TRF2 or anti-Lamin A antibody. (**F**) Sequential extraction of the nuclear matrix from HeLaI.2.11 cells. Immunoblotting was performed with anti-TRF2-2meR17, anti-TRF2, anti-Lamin A or anti-H2AX antibody. (**G**) Western analysis of early and late passage GM9503 cells. Immunoblotting was performed with anti-TRF2-2meR17 and anti-TRF2 antibody. The γ-tubulin blot was used as a loading control.

Examination of cell fractionations of hTERT-BJ with anti-Lamin A antibody revealed that Lamin A, a nuclear matrix-associated protein [38, 39], was predominantly found in the final pellet as well as fractions treated with 2M NaCl and RNase A (Fig. 1A), in agreement with these DNase I-resistant fractions being nuclear matrix. On the other hand, we found that the majority of chromatin-bound histone H2AX protein was released by the DNase I digestion (Fig. 1A), suggesting that the sequential cell fractionation protocol was working as expected. A very small amount of PRMT1 was detected in fractions associated with the nuclear matrix, consistent with previous findings [42]. In addition, a small amount of shelterin proteins including TRF1, TRF2 and hRap1 was also found in fractions associated with nuclear matrix (Fig. 1A), indicative of the nuclear matrix association of the shelterin proteins.

We have previously raised an antibody specifically against TRF2 methylated at R17 (anti-2meR17) [29], which specifically recognized methylated TRF2 (Fig. 1B) but not TRF2 carrying amino acid substitutions of arginines to lysines in its N-terminal domain (TRF2-RK) or lacking its N-terminal domain (TRF2-ΔB) (Fig. 1C). Using anti-2meR17 antibody, we found that although some methylated TRF2 was released by DNase I digestion, the majority of methylated TRF2 was recovered in nuclear matrix-associated fractions (Fig. 1A). The association of methylated TRF2 with the nuclear matrix was also observed in two other primary fibroblasts IMR90 and GM9503 cells as well as cancer cell line HeLaI.211 (Fig. 1D-1F). Taken together, these results suggest that methylated TRF2 preferentially associates with the nuclear matrix.

### Methylated TRF2 exhibits nuclear staining that is predominantly not associated with human telomeres

**Figure 2:**
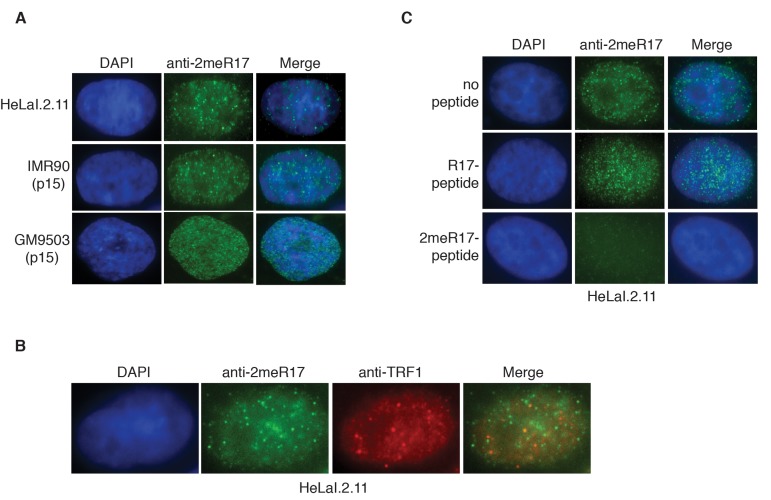
Methylated TRF2 exhibits nuclear staining largely free of human telomeres. (**A**) Analysis of indirect immunofluorescence of three different cell lines with anti-TRF2-2meR17 antibody. Cell nuclei were stained with DAPI in blue. (**B**) Analysis of dual indirect immunofluorescence with anti-TRF2-2meR17 (green) in conjunction with anti-TRF1 antibody (red). HeLaI.2.11 cell nuclei were stained in blue. (**C**) Analysis of indirect immunofluorescence with anti-TRF2-2meR17 in conjunction with 100 ng of TRF2 peptide containing either modified or unmodified arginine 17. HeLaI.2.11 cell nuclei were stained with DAPI in blue.

To further investigate the nuclear localization of methylated TRF2, we performed indirect immunofluorescence with anti-2meR17. We found that methylated TRF2 exhibited nuclear staining in both human primary (IMR90, GM9503) and cancer (HeLaI.2.11) cells (Fig. 2A). Analysis of dual indirect immunofluorescence with anti-2meR17 in conjunction with antibody against TRF1 [43], a marker for interphase telomeres, revealed that although there appeared to be some overlap between anti-2meR17 staining and anti-TRF1 staining (Fig. 2B), the majority of methylated TRF2 was not found to localize at telomeres (Fig. 2B). To investigate whether the observed anti-2meR17 staining might be due to any non-specific binding, we performed indirect immunofluorescence with anti-2meR17 antibody in the presence of TRF2 peptide containing either unmodified R17 or dimethylated R17.

We found that while TRF2 peptide containing unmodified R17 had little effect on the nuclear staining of methylated TRF2 in these cell lines (Fig. 2C and data not shown), TRF2 peptide containing dimethylated R17 abrogated the nuclear staining of methylated TRF2 (Fig. 2C and data not shown). These results suggest that the observed anti-2meR17 staining is unlikely due to non-specific binding. Taken together, these results suggest that methylated TRF2 localizes in nuclear domains largely free of human telomeres. These results are in agreement with our previous report that arginine methylation negatively regulates TRF2 association with telomere chromatin [29].

### Replicative senescence induces altered nuclear staining of methylated TRF2

**Figure 3:**
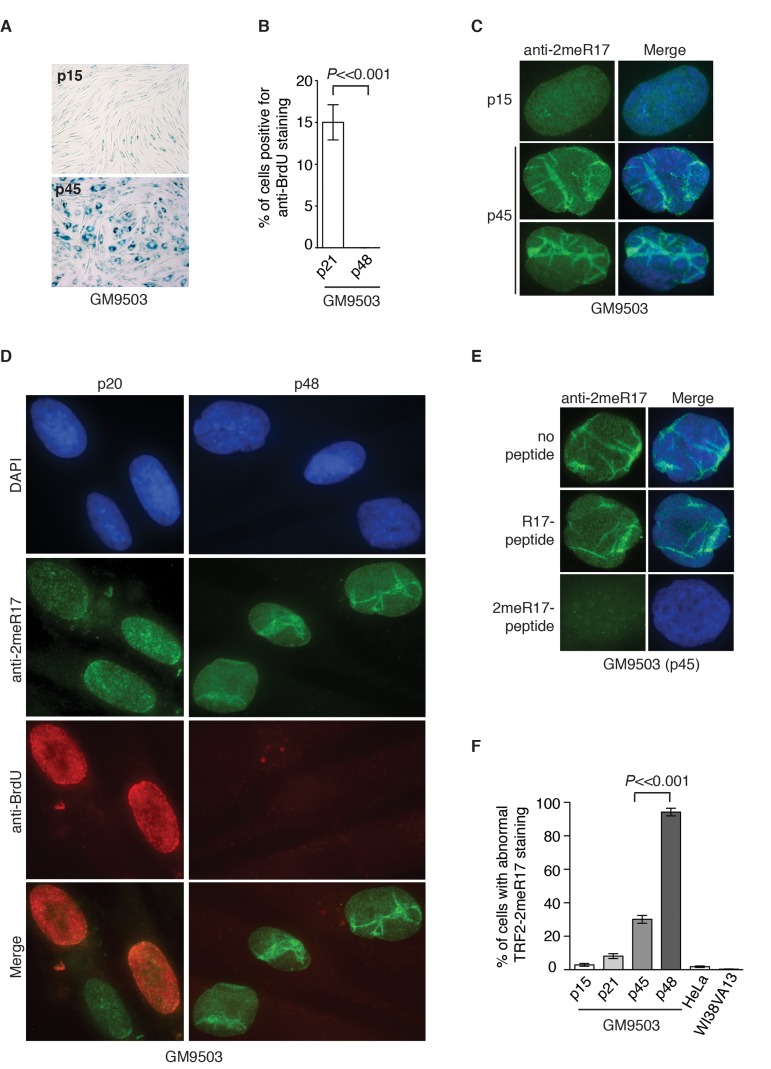
Methylated TRF2 exhibits an altered nuclear staining associated with induction of replicative senescence in normal human primary fibroblast GM9503 cells. (**A**) Senescence-associated β-galactosidase assays for GM9503 cells at either p15 or p45. (**B**) Quantification of percentage of young and senescent GM9503 cells with BrdU incorporation. A total of 300 cells in triplicate were scored for either early passage (p21) or senescent GM9503 (p48) cells. Standard deviations from three independent experiments are indicated. (**C**) Analysis of indirect immunofluorescence with anti-TRF2-2meR17 antibody in GM9503 cells at either p15 or p45. Cell nuclei were stained with DAPI in blue. (**D**) Analysis of dual indirect immunofluorescence with anti-TRF2-2meR17 antibody (green) in conjunction with anti-BrdU antibody (red). The early passage (p20) and senescent GM9503 (p48) cells were incubated for six hours in growth media containing 10 μM BrdU prior to being processed for immunofluorescence. Cell nuclei were stained with DAPI in blue. (**E**) Analysis of indirect immunofluorescence with anti-TRF2-2meR17 in conjunction with 100 ng of TRF2 peptide containing either modified or unmodified arginine 17. Cell nuclei were stained with DAPI in blue. (**F**) Quantification of percentage of cells with altered nuclear staining of methylated TRF2. At least 900 cells in triplicate were scored in blind for each transformed cell line or each normal primary fibroblast cell line at a given passage as indicated. Both HeLa and WI38VA13 are transformed cell lines. Standard deviations from three independent experiments are indicated.

We have shown that methylated TRF2 is associated with the nuclear matrix, which is known to undergoalterations in the process of replicative senescence [44]. To investigate whether the association of methylated TRF2 with the nuclear matrix might be affected by cellular senescence, we performed the sequential extraction of nuclear matrix in both early and late passages of primary skin fibroblast GM9503 cells. GM9503 cells exhibited an accumulation of senescent cells at passage 45 (Fig. 3A). Analysis of cell fractionations with anti-2meR17 antibody revealed that the association of methylated TRF2 with the nuclear matrix in late passage GM9503 (p45) cells was indistinguishable from that in young GM9503 (p21) cells (Fig. 1E). We did not observe any significant change in the level of methylated TRF2 and the total TRF2 between early and late passages of GM9503 cells (Fig. 1G). Taken together, these results suggest that methylated TRF2 does not dissociate from the nuclear matrix during the process of cellular senescence.

To investigate whether the nuclear staining of methylated TRF2 might undergo any changes during the process of replicative senescence, we performed indirect immunofluorescence with anti-2meR17 antibody in GM9503 cells of various passages from young to senescent. At passage 45, the majority of GM9503 cells were found to be positive for senescence-associated β-galactosidase staining (Fig. 3A). After they reached passage 48, GM9503 cells appeared to have fully entered replicative senescence since they failed to gain one population doubling over a period of at least two weeks and they showed little incorporation of BrdU (Fig. 3B), a marker used to measure DNA synthesis in cycling cells.

We found that methylated TRF2 showed a rather homogenous nuclear staining in young GM9503 (p15 and p20) cells (Fig. 3C and 3D), however, its staining was drastically altered in late passage and senescent GM9503 (p45 and p48) cells, (Fig. 3C and 3D and Supplementary Fig. 1). To address a concern that this altered nuclear staining might be due to any non-specific binding of anti-2meR17 antibody, we performed indirect immunofluorescence with anti-2meR17 antibody in the presence of TRF2 peptide containing either unmodified R17 or dimethylated R17. We found that TRF2 peptide containing unmodified R17 had little effect on the altered nuclear staining of methylated TRF2 whereas TRF2 peptide containing dimethylated R17 abolished TRF2 staining in senescent cells (Fig. 3E), arguing against the possibility that the observed altered nuclear staining of methylated TRF2 is due to non-specific binding of the anti-2meR17 antibody.

While less than 3% of young GM9503 (p15) cells displayed the altered methylated TRF2 staining (Fig. 3F), about 30% of late passage GM9305 (p45) cells showed the altered methylated TRF2 staining (Fig. 3F). To investigate whether the altered nuclear staining of methylated TRF2 might occur in non-dividing cells, we cultured GM9503 cells in media containing BrdU. Analysis of dual indirect immunofluorescence with anti-2meR17 antibody in conjunction with anti-BrdU antibody revealed that the altered methylated TRF2 staining was always associated with nuclei lacking incorporation of BrdU (Fig. 3D). We found that in the senescent GM9503 (p48) cell culture with little BrdU incorporation (Fig. 3B), over 90% of nuclei exhibited the altered methylated TRF2 staining (Fig. 3F). Taken together, these results suggest that the altered methylated TRF2 staining is associated with replicative senescence in GM9503 cells.

**Figure 4:**
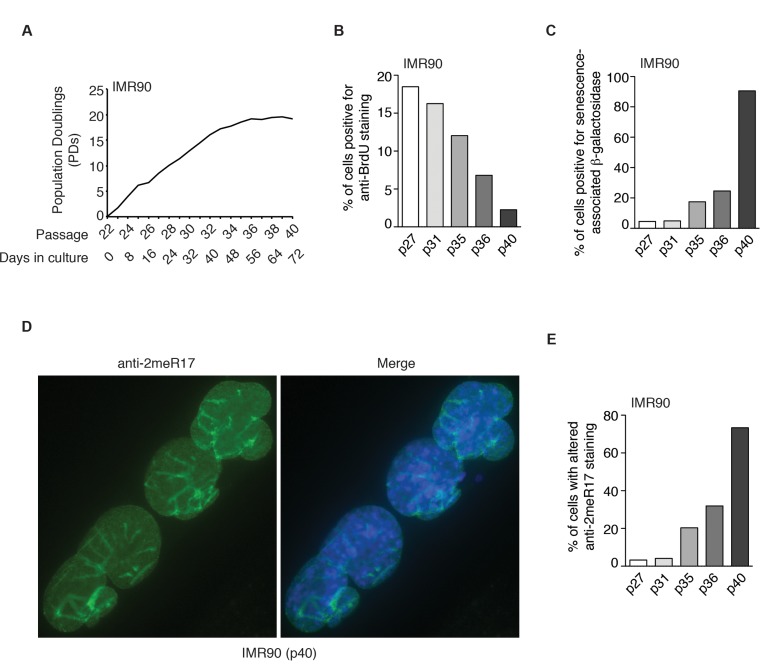
The altered nuclear staining of methylated TRF2 is a general characteristics of replicative senescence. (**A**) Growth curve of IMR90 cells. IMR90 cells (p22) were passaged every four days continously for 72 days. (**B**) Quantification of percentage of IMR90 cells with BrdU incorporation. A total number of 952, 1027, 1146, 1720 and 1418 cells were scored for passages 27, 31, 35, 36 and 40, respectively. (**C**) Quantification of percentage of IMR90 cells with senescence-associated β-galactosidase staining. A total number of 1887, 1606, 1286, 1712 and 1047 cells were scored for passages 27, 31, 35, 36 and 40, respectively. (**D**) Analysis of indirect immunofluorescence with anti-2meR17 antibody in senescent IMR90 (p40) cells. Cell nuclei were stained with DAPI in blue. (**E**) Quantification of percentage of IMR90 cells with altered nuclear staining of methylated TRF2. A total number of 952, 1027, 1146, 1720 and 1418 cells were scored for passages 27, 31, 35, 36 and 40, respectively.

We also observed an induction of the altered nuclear staining of methylated TRF2 in several other normal human primary fibroblast cell lines (AG02661 and GM1706) as they aged in cultures (Supplementary Fig. 2). To further investigate whether the altered methylated TRF2 staining might be a general feature of replicative senescence, we passaged young IMR90 (p22) cells continuously every four days for 72 days until they reached passage 40 (Fig. 4A). In the last two weeks of culturing, IMR90 failed to gain one population doubling (Fig. 4A), suggesting that the IMR90 cell culture had entered replicative senescence. In agreement with this notion, we found that only 2% of IMR90 (p40) cells were stained positive for anti-BrdU antibody (Fig. 4B) and that about 90% of IMR90 cells were stained positive for senescence-associated β-galactosidase (Fig. 4C). Analysis of indirect immunofluorescence with anti-2meR17 antibody revealed that the number of IMR90 cells with the altered methylated TRF2 staining increased substantially as IMR90 cells aged in culture (Fig. 4D and 4E). While less than 4% of IMR90 (p27) cells showed the altered nuclear staining of methylated TRF2, the altered methylated staining was observed in over 73% of senescent IMR90 (p40) cells (Fig. 4E). On the other hand, we did not detect any significant accumulation of the altered staining of methylated TRF2 in cancer and transformed cell lines including HeLa and WI38VA13/2RA (Fig. 3F). Collectively, these results suggest that the altered nuclear staining of methylated TRF2 is a general characteristic associated with replicative senescence.

### Altered nuclear staining of methylated TRF2 is associated with the altered nuclear structure in senescent cells

**Figure 5:**
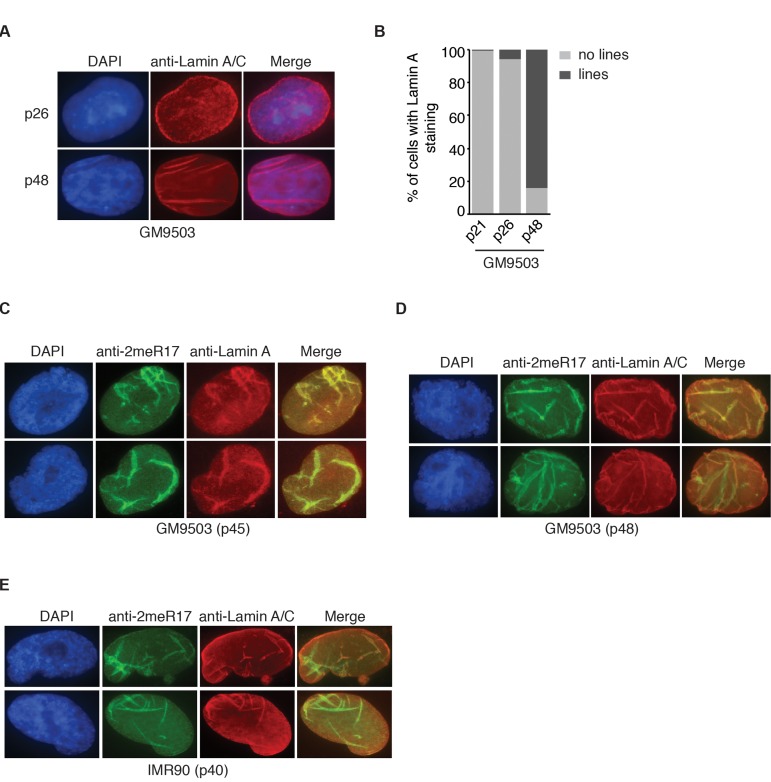
The altered nuclear staining of methylated TRF2 is associated with altered nuclear structure in senescent cells. (**A**) Analysis of indirect immunofluorescence with anti-Lamin A/C antibody on young (p26) and senescent GM9503 (p48) cells. Cell nuclei were stained with DAPI in blue. (**B**) Quantification of percentage of GM9503 cells showing nuclear Lamin A staining that are either line-shaped or not associated with lines. A total number of 1014, 1050 and 1073 cells were scored for passages 21, 26 and 48, respectively. (**C**) Analysis of dual indirect immunofluorescence in late passage GM9503 (p45) with anti-TRF2-2meR17 antibody in conjunction with anti-Lamin A antibody. Cell nuclei were stained with DAPI in blue. (**D**) Analysis of dual indirect immunofluorescence in senescent GM9503 (p48) with anti-TRF2-2meR17 antibody in conjunction with anti-Lamin A/C antibody. Cell nuclei were stained with DAPI in blue. (**E**) Analysis of dual indirect immunofluorescence in senescent IMR90 (p40) with anti-TRF2-2meR17 antibody in conjunction with anti-Lamin A/C antibody. Cell nuclei were stained with DAPI in blue.

It has been reported that senescent and aged cells are associated with distorted nuclear defects [11, 12, 44]. We examined 1182 senescent GM9503 (p48) cells and found that over 96% of the nuclei with altered methylated TRF2 staining contained blebbings and/or herniations, suggesting that the altered methylated TRF2 staining is overwhelmingly associated with misshapen nuclei. Lamin A has been suggested to serve as a marker for distorted nuclear defects in aged cells [11, 12] and thus we also examined the relationship between the altered methylated TRF2 staining and Lamin A staining in senescent GM9503 (p48) cells. In young GM9503 (p21) cells, we observed strong Lamin A staining associated with the nuclear periphery as well as less intense Lamin A staining in the nucleoplasm (Fig. 5A). However, as cells aged in culture, we observed the appearance of line-shaped Lamin A staining in the nucleus. We found that in the senescent GM9503 (p48) culture, over 85% of cells exhibited line-shaped Lamin A/C staining (Fig. 5B). Analysis of dual indirect immunofluorescence with anti-2meR17 antibody in conjunction with either anti-Lamin A or anti-Lamin A/C antibody revealed that the line-shaped Lamin A staining overlapped well with the altered methylated TRF2 staining in GM9503 cells (Fig. 5C and 5D). The overlap between the altered nuclear staining of methylated TRF2 and the line-shaped Lamin A staining was also observed in senescent IMR90 (p40) cells (Fig. 5E). We examined 1073 nuclei of senescent GM9503 (p48) cells and found that 88% of nuclei had both the altered methylated TRF2 staining and the line-shaped Lamin A staining. Taken together, these results suggest that the altered nuclear staining of methylated TRF2 is predominantly associated with senescence-induced distorted nuclear structure.

### Overexpression of hTERT prevents the formation of senescence-induced altered nuclear staining of methylated TRF2

**Figure 6:**
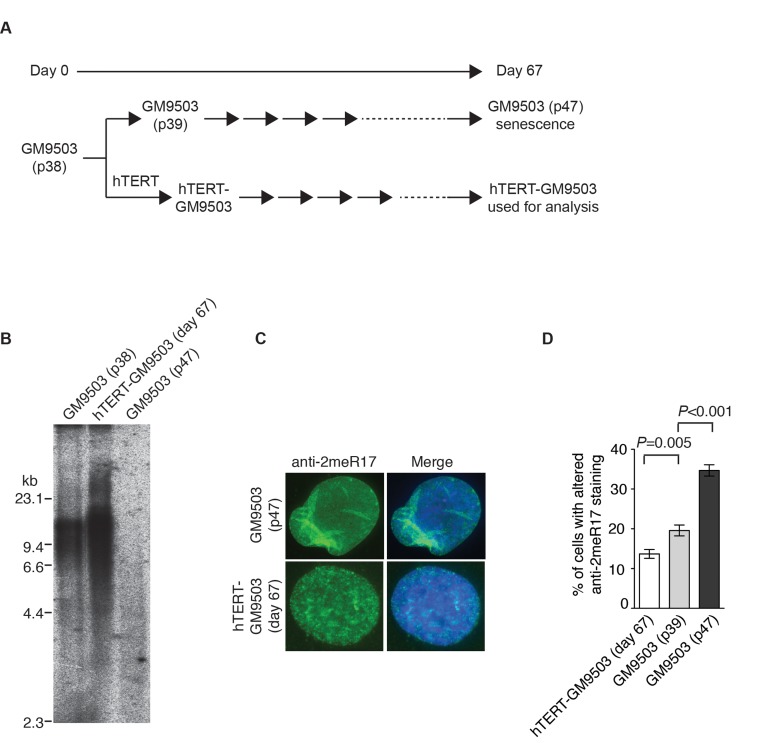
Introduction of hTERT into normal primary fibroblast cells suppresses the formation of senesence-associated altered nuclear staining of methylated TRF2. (**A**) Schematic diagram of the experimental setup. At day 0, GM9503 cells were infected with retrovirus expressing hTERT, generating hTERT-GM9503 cells. Both GM9503 and hTERT-GM9503 cells were cultured continuously for 67 days. (**B**) Genomic blots of telomeric restriction fragments from GM9503 (p38), GM9503 (p47) and hTERT-GM9503 at day 67. About 3 μg of RsaI/HinfI-digested genomic DNA from each sample was used for gel electrophoresis. The DNA molecular size markers are shown to the left of the blots. (**C**) Analysis of indirect immunofluorescence with anti-TRF2-2meR17 antibody. Cell nuclei of GM9503 and hTERT-GM9503 were stained with DAPI in blue. (**D**) Quantification of percentage of cells with altered nuclear staining of methylated TRF2. At least 900 cells in triplicate were scored in blind for each cell line as indicated. Standard deviations from three independent experiments are indicated.

Overexpression of hTERT can prevent replicative senescence, resulting in immortalization of normal primary fibroblasts [45, 46]. To investigate whether hTERT may repress the formation of the altered nuclear staining of methylated TRF2, we introduced hTERT into GM9503 cells of passage 38 (p38) and cultured hTERT-GM9503 cells in parallel with parental GM9503 cells (p38) until GM9503 cells approached replicative senescence at p47 (Fig. 6A). Exogenous expression of hTERT was sufficient to immortalize GM9503 cells and prevented telomere shortening associated with replicative senescence (Fig. 6B). Analysis of indirect immunofluorescence with anti-2meR17 antibody revealed an induction of an altered nuclear staining of methylated TRF2 in GM9503 cells at passage 47 compared to those at passage 39 (Fig. 6C and 6D), consistent with our earlier finding. On the other hand, no increase in the altered nuclear staining of methylated TRF2 was observed in hTERT-immortalized GM9503 cells. Instead, we detected a 30% decrease (*P*=0.005) in the number of hTERT-GM9503 cells with an altered nuclear staining of methylated TRF2 when compared to GM9503 cells of passage 39 (Fig. 6D). These results suggest that overexpression of hTERT suppresses the formation of replicative senescence-induced altered nuclear staining of methylated TRF2.

### Dysfunctional telomeres induce the formation of the altered nuclear staining of methylated TRF2

**Figure 7:**
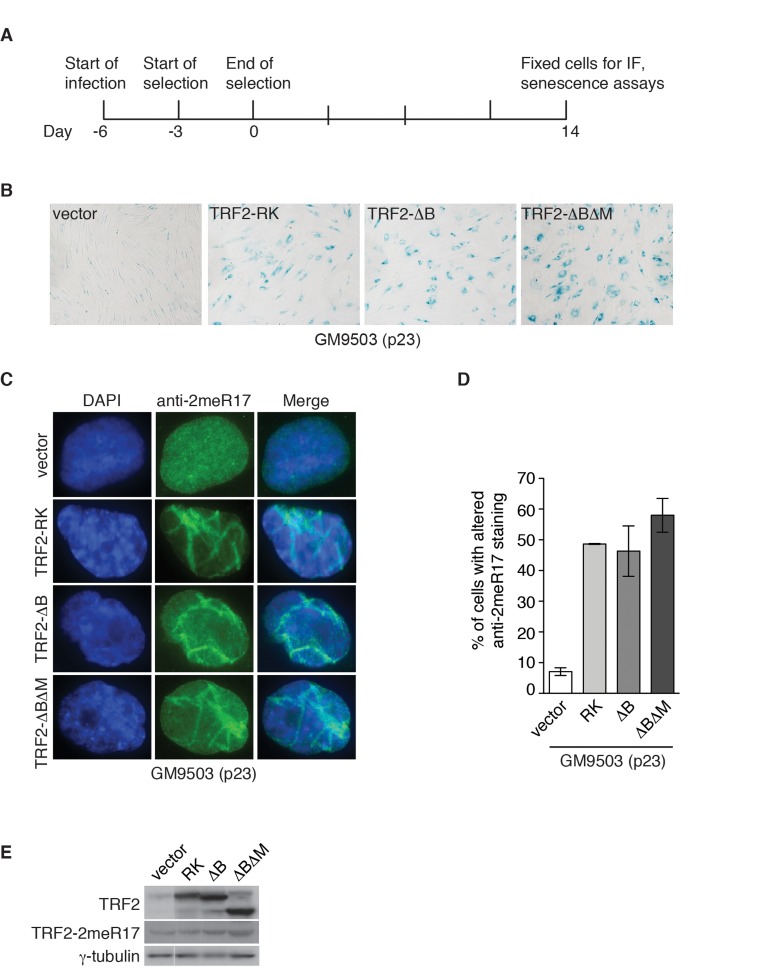
Dysfunctional telomeres induce altered nuclear staining of methylated TRF2. (**A**) Schematic diagram of experimental setup. GM9503 cells (p23) were infected with retrovirus expressing various TRF2 mutant alleles at day -6. After the three-day selection ended on day 0, the cells were cultured for 14 days and then subjected to analysis of immunofluorescence (IF) and cellular senescence. (**B**) Senescence-associated β-galactosidase assays of GM9503 cells overexpressing the vector alone or various TRF2 mutant alleles as indicated. (**C**) Indirect immunofluorescence with anti-TRF2-2meR17 antibody in fixed GM9503 cells overexpressing the vector alone or various TRF2 mutant alleles as indicated. Cell nuclei were stained with DAPI in blue. (**D**) Quantification of percentage of cells with altered nuclear staining of methylated TRF2. A total of 1000 cells in triplicate were scored in blind for each cell line. Standard deviations from three independent experiments are indicated. (**E**) Western analysis of GM9503 cells overexpressing various TRF2 mutant alleles as indicated. Immunoblotting was carried out with anti-TRF2, anti-TRF2-2meR17 or anti-γ-tubulin antibody.

In addition to programmed telomere shortening, dysfunctional telomeres resulting from disruption of TRF2 function can also induce cellular senescence [26, 28, 29]. It has been well documented that overexpression of TRF2 lacking the N-terminal basic/GAR domain (TRF2-ΔB) promotes telomere rapid deletion whereas overexpression of TRF2 lacking both the N-terminal basic/GAR and C-terminal Myb-like DNA binding domains (TRF2-ΔBΔM) induces the formation of telomere fusions, both of which result in the induction of cellular senescence [26, 28]. Overexpression of TRF2 carrying amino acid substitutions of arginines to lysines in its N-terminal domain (TRF2-RK) has been shown to induce fragile telomeres, triggering cellular senescence [29]. To investigate whether the altered nuclear staining of methylated TRF2 might be associated with cellular senescence induced by dysfunctional telomeres, we infected GM9503 (p23) cells with retrovirus expressing TRF2-RK, TRF2-ΔB, TRF2-ΔBΔM or the vector alone. Fourteen days post infection (Fig. 7A), we observed an induction of cellular senescence in GM9503 cells overexpressing either TRF2-RK, TRF2-ΔB or TRF2-ΔBΔM but not in GM9503 cells expressing the vector alone (Fig. 7B), consistent with previous reports [26, 28, 29]. Analysis of indirect immunofluorescence revealed a drastic accumulation of the altered nuclear staining of methylated TRF2 in GM9503 cells overexpressing either TRF2-RK, TRF2-ΔB or TRF2-ΔBΔM when compared to GM9503 cells expressing the vector alone (Fig. 7C and 7D). Overexpression of TRF2-RK, TRF2-ΔB or TRF2-ΔBΔM had little effect on the level of endogenous methylated TRF2 (Fig. 7E). These results suggest that the altered nuclear staining of methylated TRF2 is associated with cellular senescence induced by dysfunctional telomeres.

### DNA damage induces the formation of the altered nuclear staining of methylated TRF2 in an ATM-dependent manner

**Figure 8:**
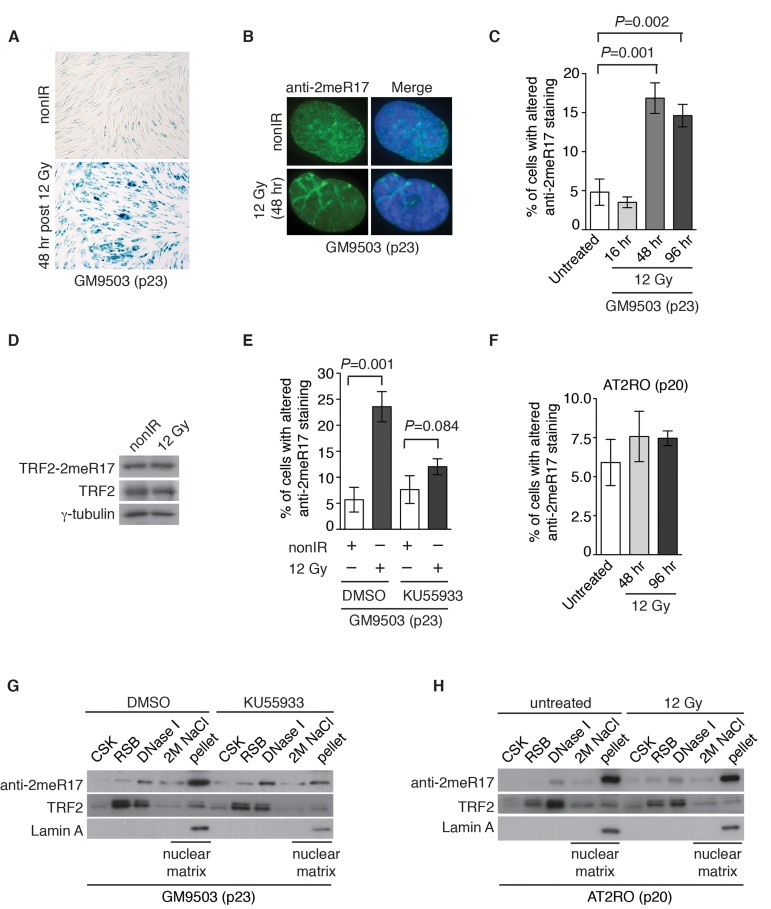
Ionizing radiation induces altered nuclear staining of methylated TRF2 in a ATM-dependent manner. (**A**) Ionizing radiation induces cellular senescence in GM9503 cells. GM9503 (p23) cells were treated with 12 Gy IR. Senescence-associated β-galactosidase assays were performed 48 h post IR. (**B**) Indirect immunofluorescence with anti-TRF2-2meR17 antibody in mock- or IR-treated GM9503 cells (p23). Cell nuclei were stained with DAPI in blue. (**C**) Quantification of percentage of cells with altered nuclear staining of methylated TRF2. A total of 1000 cells in triplicate were scored in blind for untreated or IR-treated cells fixed at various time points post IR as indicated. Standard deviations from three independent experiments are indicated. (**D**) Western analysis of GM9503 cells (p23) that were either mock- or IR-treated. Immunoblotting was performed with anti-TRF2-2meR17 or anti-TRF2 antibody. The γ-tubulin blot was used as a loading control. (**E**) ATM inhibition abrogates IR-induced altered nuclear staining of methylated TRF2. GM9503 cells (p23) were treated with DMSO or KU55933 prior to 12 Gy IR treatment. Forty-eight hours post IR, cells were processed for indirect immunofluorescence with anti-TRF2-2meR17 antibody. Quantification of percentage of cells with altered nuclear staining of methylated TRF2. A total of 1000 cells in triplicate were scored in blind. Standard deviations from three independent experiments are indicated. (**F**) Little IR-induced altered nuclear staining of methylated TRF2 is observed in AT2RO cells lacking functional ATM. Quantification of percentage of cells with altered nuclear staining of methylated TRF2. A total of 1000 cells in triplicate were scored in blind. Standard deviations from three independent experiments are indicated. (**G**) Sequential extraction of the nuclear matrix from GM9503 cells treated with either DMSO or KU55933. Immunoblotting was performed with anti-TRF2-2meR17, anti-TRF2 or anti-Lamin A antibody. (**H**) Sequential extraction of the nuclear matrix from AT2RO cells that were either untreated or treated with 12 Gy IR. Immunoblotting was performed with anti-TRF2-2meR17, anti-TRF2 or anti-Lamin A antibody.

Stress induced premature senescence is known to be a DNA damage response [47] and therefore we asked whether the altered nuclear staining of methylated TRF2 might be associated with DNA damage-induced senescence. To address this question, GM9503 cells (p23) were either mock-treated or treated with 12 Gy ionizing irradiation (IR). We found that 48 h post IR, all GM9503 cells entered cellular senescence as evidenced by analysis of senescence-associated β-galactosidase assays (Fig. 8A). Analysis of indirect immunofluorescence with anti-2meR17 revealed an over 3-fold induction in the number of GM9503 cells with the altered staining of methylated TRF2 48 h post 12 Gy IR (Fig. 8B and 8C). We did not detect any significant change in the level of methylated TRF2 (Fig. 8D). Collectively, these results suggest that the altered nuclear staining of methylated TRF2 is associated with DNA damage-induced cellular senescence.

ATM is a master regulator of DNA damage response following ionizing radiation [40, 41] and therefore we also examined whether IR-induced altered nuclear staining of methylated TRF2 might be dependent upon the ATM-mediated DNA damage response. GM9503 cells (p23) were treated with DMSO or KU55933, a potent and specific ATM inhibitor [48], prior to 12 Gy IR. Analysis of indirect immunofluorescence with anti-2meR17 antibody revealed that treatment with KU55933 impaired the induction of IR-induced altered nuclear staining of methylated TRF2 (Fig. 8E). The impairment of IR-induced altered nuclear staining of methylated TRF2 was also observed in primary ataxia telangiectasia (AT) fibroblast cells (AT2RO) lacking functional ATM (Fig. 8F). Treatment with KU55933 or loss of ATM did not affect the association of methylated TRF2 with the nuclear matrix (Fig. 8G and H). Taken together, these results suggest that ATM is important for the formation of IR-induced altered nuclear staining of methylated TRF2.

## DISCUSSION

In this report, we have shown that methylated TRF2 is associated with the nuclear matrix and that this localization is largely free of human telomeres. In addition we have uncovered that the nuclear staining of methylated TRF2 is drastically altered upon induction of cellular senescence and that this altered staining is predominantly associated with misshapen nuclei and abnormal nuclear matrix folds. Our findings suggest that methylated TRF2 can serve as a potential biomarker for cellular senescence.

Through sequential extraction of the nuclear matrix, we have shown that the nuclear matrix contains a small amount of endogenous TRF1, TRF2 and hRap1, suggesting that components of the shelterin complex are associated with the nuclear matrix, in agreement with previous reports [10, 17]. We have previously estimated that about 1-5% of endogenous TRF2 is methylated by PRMT1 [29]. Our finding that the majority of methylated TRF2 is associated with the nuclear matrix suggests that arginine methylation plays an important role in regulating TRF2 interaction with the nuclear matrix.

We have previously reported that overexpression of TRF2-RK carrying amino acid substitutions of arginines to lysines (TRF2-RK), which cannot undergo PRMT1-dependent arginine methylation [29], promotes the formation of telomeres with multiple telomere signals [29], also known as fragile telomeres [49, 50]. Our finding that methylated TRF2 is associated with the nuclear matrix raises a possibility that TRF2-RK-induced fragile telomeres might arise in part from its inability to interact with the nuclear matrix. As fragile telomeres are thought to result from a defect in telomere replication [49, 50], it would be of interest to know if the arginine methylation-dependent TRF2 interaction with the nuclear matrix might play a role in supporting efficient telomere replication.

Although we have shown that methylated TRF2 co-fractionates with Lamin A, we have not been able to detect a direct interaction between TRF2 and Lamin A (T. Mitchell and X.-D. Zhu, unpublished data), suggesting that the association of methylated TRF2 with the nuclear matrix may not be mediated by Lamin A. While human telomeres are attached to the nuclear matrix [16], we have shown through indirect immunofluorescence that the punctate nuclear staining of methylated TRF2 is largely free of telomere signals, suggesting that methylated TRF2 interaction with the nuclear matrix is unlikely mediated through telomeric DNA. How methylated TRF2 interacts with the nuclear matrix remains unknown, but our finding nevertheless suggests that post-translational modification plays a role in regulating TRF2 interaction with the nuclear matrix. Future studies would be needed to investigate the nature of the punctate nuclear staining of methylated TRF2 observed in young primary cells and cancer cells.

We have shown that methylated TRF2 exhibits an altered nuclear staining upon induction of replicative senescence in normal human primary fibroblasts and that this altered staining can be suppressed by overexpression of hTERT, suggesting that telomere erosion may be a trigger for the altered nuclear staining of methylated TRF2. It has been suggested that ionizing radiation (IR)- and oncogene-induced cellular senescence is caused by irreparable DNA damage at telomeres [51, 52]. Our observation that the altered nuclear staining of methylated TRF2 is associated with IR- and dysfunctional telomeres-induced cellular senescence further supports the notion that telomere damage resulting from either genotoxic insults or programmed telomere erosion is a major inducer of the altered nuclear staining of methylated TRF2.

Using two independent antibodies against Lamin A, we have observed that senescent fibroblast cells (GM9503 and IMR90) exhibit abnormal line-shaped Lamin A staining, similar to the Lamin A staining previously reported for senescent human mesenchymal stem cells [11, 44]. Our finding that the altered nuclear staining of methylated TRF2 overlaps well with the abnomal line-shaped Lamin A staining suggests that the altered methylated TRF2 staining is associated with distorted nuclear structures in senescent cells. Our finding further implies that methylated TRF2 may serve as a potential biomarker for senescence-associated altered nuclear matrix.

It has been well established that the ATM-dependent DNA damage response is needed for initiating and maintaining cellular senescence [53-56]. Consistent with previous reports [53-56], we have observed that primary AT2RO fibroblasts lacking functional ATM fail to undergo cell cycle arrest and start to die within days after ionizing radiation (T.R.H Mitchell and X.-D. Zhu, unpublished data). We have demonstrated that loss or inhibition of ATM abrogates IR-induced altered nuclear staining of methylated TRF2, indicating that the ATM-dependent DNA damage response is needed for the formation of the altered nuclear staining of methylated TRF2. Our finding suggests that the methylated TRF2 may serve as a potential biomarker for aging- and stress-induced cellular senescence.

## METHODS

### DNA constructs and antibodies

The retroviral construct expressing shRNA against PRMT1 (pRS-shPRMT1) has been previously described [29]. The retroviral construct expressing hTERT (pBabe-hTERT) was generously provided by Robert Weinberg, Whitehead Institute for Biomedical Research.

Antibody specifically raised against TRF2 methylated at R17 (2meR17) has been previously described [29]. Antibodies against TRF1, TRF2 and hRap1 were kind gifts from Titia de Lange, Rockefeller University. Other antibodies used include Lamin A (Millipore), Lamin A/C (Cell Signaling), PRMT1 (Millipore) and H2A.X (Upstate).

### Cell culture and retroviral infection

Cells were grown in DMEM medium with 10% fetal bovine serum (FBS) for HeLaI.2.11, 293T, WI38VA13/2RA, hTERT-BJ and Phoenix cells supplemented with non-essential amino acids, L-glutamine, 100 U/ml penicilin and 0.1 mg/ml streptomycin. Supplementary DMEM medium plus 15% FBS was used to culture normal primary human fibroblasts (IMR90, GM9503, AG02261 and GM1706) (Coriell) and ATM-deficient primary fibroblast AT2RO (a kind gift from Jan Hoeijmakers). For inhibition of ATM, cells were treated with KU55933 (20 μM, Sigma) for 3 h before 12 Gy ionizing irradiation treatment. Ionizing irradiation was delivered from a Cs-137 source at McMaster University (Gammacell 1000).

Retroviral gene delivery was carried out essentially as described [29, 57]. Phoenix amphotropic retroviral packaging cells were transfected with the desired DNA constructs using Lipofectamine 2000 (Invitrogen) according to the manufacturer. At 36, 48, 60, 72, and 84 h post-transfection, the virus-containing medium was collected and used to infect cells in the presence of polybrene (4 μg/ml). Twelve hours after the last infection, puromycin (2 μg/ml) was added to the medium, and the cells were maintained in the selection media for the entirety of the experiments.

### Sequential extraction of nuclear matrix

Extraction of nuclear matrix components was conducted essentially as described [58, 59]. Briefly, PBS-washed cells were resuspended in 5X pellet volume cytoskeleton (CSK) buffer (10 mM Pipes pH 6.8, 100 mM NaCl, 300 mM sucrose, 3 mM MgCl_2_, 1 mM EGTA, 2 mM vanadyl ribonucleoside complex, 1.2 mM phenylmethylsulfonyl fluoride, 1 mM DTT, 1 μg/ml aprotinin, 1 μg/ml leupeptin, 10 μg/ml pepstatin and 0.5% Triton X-100). Following centrifugation at 1000g for 5 min, the cytoskeleton framework was further extracted by incubating the pellet in RSB-magik buffer (10 mM Tris-HCl pH 7.4, 10 mM NaCl, 3 mM MgCl_2_, 2 mM vanadyl ribonucleoside complex, 1.2 mM phenylmethylsulfonyl fluoride, 1 mM DTT, 1 μg/ml aprotinin, 1 μg/ml leupeptin, 10 μg/ml pepstatin, 1% Tween 20 and 0.5% sodium deoxycholate) for 5 min. Upon centrifugation, the pellet was treated with 30-50 U of RNase-free DNaseI (Fermentas) per 10^6^ cells in digestion buffer (10 mM Pipes pH6.8, 50 mM NaCl, 300 mM sucrose, 3 mM MgCl_2_, 1 mM EGTA, 2 mM vanadyl ribonucleoside complex, 1.2 mM phenylmethylsulfonyl fluoride, 1 mM DTT, 1 μg/ml aprotinin, 1 μg/ml leupeptin, 10 μg/ml pepstatin and 0.5% Triton X-100) for 1 h at room temperature. Chromatin was then removed by elution with 0.25 M ammonium sulfate, leaving a complete nuclear matrix-intermediate filament scaffold containing nuclear ribonuclear-protein complexes [58]. The complete nuclear matrix was further extracted with 2M NaCl to release the outer nuclear matrix proteins, and in some cases followed by digestion with DNase-free RNase A to remove the core filaments of the matrix. All incubation and centrifugations were performed at 4°C except where indicated.

### Immunofluorescence

Immunofluorescence was performed as described [29, 60, 61]. All cell images were recorded on a Zeiss Axioplan 2 microscope with a Hammamatsu C4742-95 camera and processed using the Openlab software package.

For BrdU labeling, cells were seeded on and processed in 8-well chamber slides (Lab Tek). Two days later, cells were incubated for 6 hours in media containing 10 μM BrdU (Sigma) and then fixed at room temperature (RT) for 7 min in PBS-buffered 3% paraformaldehyde and 2% sucrose. Following permeablization at RT for 7 min in Triton X-100 buffer (0.5% Triton X-100, 20 mM Hepes-KOH, pH 7.9, 50 mM NaCl, 3 mM MgCl_2_, 300 mM sucrose), cells were incubated in 4M HCl for 10 min at RT. Cells were then processed for immunofluorescence as described [29,60, 61] using anti-BrdU antibody (Novus Biologicals) and anti-2meR17 antibody.

### Telomere length analysis and senescence-associated β-galactosidase assays

For telomere length analysis, genomic DNA isolated from cells was digested with *Rsa*I and *Hinf*I and loaded onto a 0.7% agarose gel in 0.5xTBE. Blotting for telomeric fragments was carried out as described [62, 63].

Senescence-associated (SA) β-galactosidase assays were carried out using the SA-β-gal senescence kit (Cell Signaling) according to the manufacturer's instructions. The cells were seeded two to four days prior to processing.

### Statistical analysis

A student's two-tailed t test was used to derive all *P* values.

## SUPPLEMENTAL FIGURES


