# Supplementary material for: Methylated TRF2 associates with the nuclear matrix and serves as a potential biomarker for cellular senescence
Source: Aging (Albany NY). 2014 Apr 5;6(4):248–63. doi: 10.18632/aging.100650 (PMC4032793; doi:10.18632/aging.100650)
Supplement: Supplementary file 1 [file aging-06-248-s001.pdf]

**61.** McKerlie M and Zhu XD. Cyclin B-dependent kinase 1 regulates human TRF1 to modulate the resolution of sister telomeres. Nat Commun. 2011; 2:371 doi: 10.1038/ncomms1372.

**62.** Wu Y, Zacal NJ, Rainbow AJ and Zhu XD. XPF with mutations in its conserved nuclease domain is defective in DNA repair but functions in TRF2-mediated telomere shortening. DNA Repair (Amst). 2007; 6:157-166.

**63.** Wu Y, Xiao S and Zhu XD. MRE11-RAD50-NBS1 and ATM function as co-mediators of TRF1 in telomere length control. Nat Struct Mol Biol. 2007; 14:832-840.

## SUPPLEMENTARY FIGURES

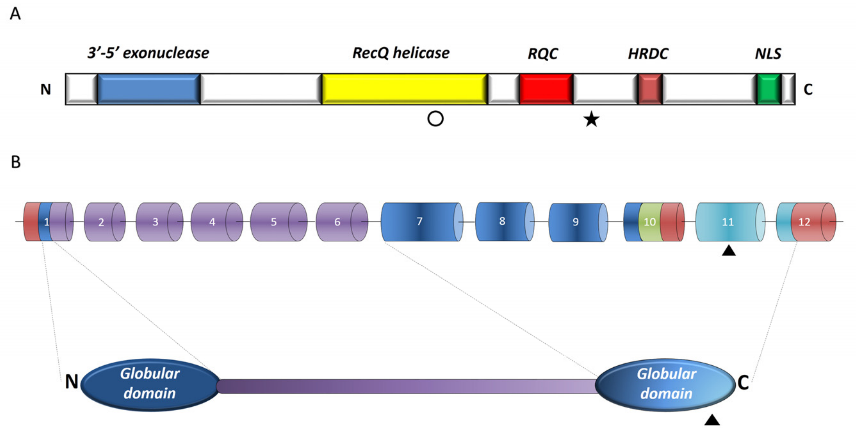


**Supplementary Figure 1**. Methylated TRF2 exhibits an altered nuclear staining associated with induction of replicative senescence in normal human primary fibroblast cells. Analysis of indirect immunofluorescent with anti-TRF2-2meR17 antibody. Cell nuclei were stained with DAPI in blue.

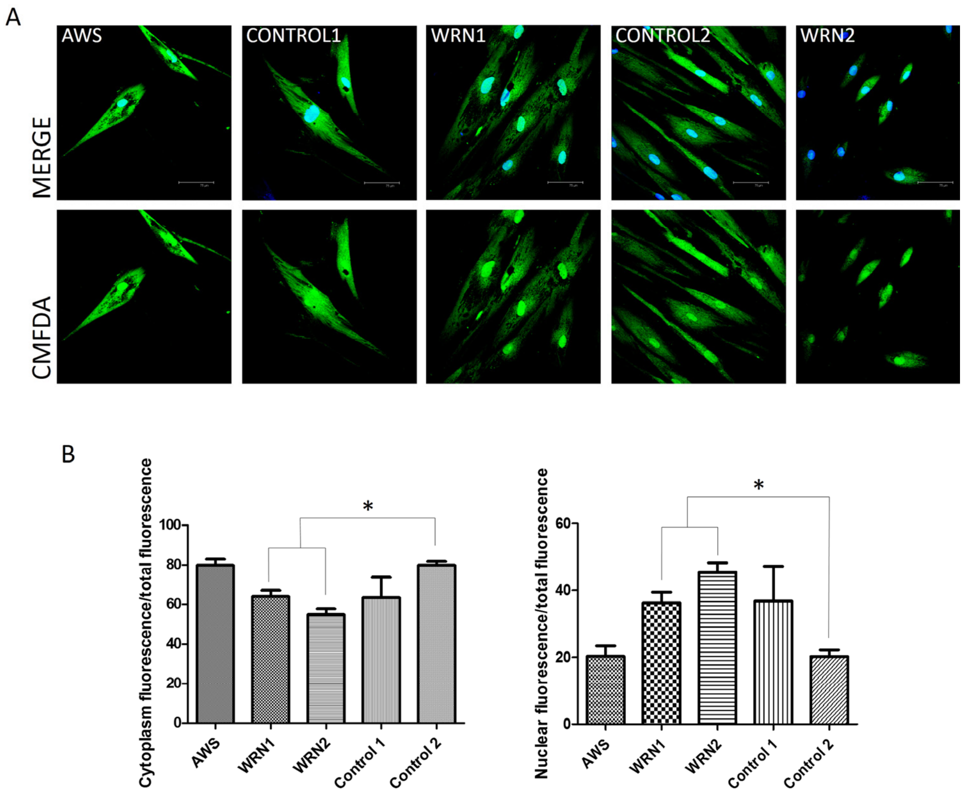


**Supplementary Figure 2**. Induction of the altered methylated TRF2 staining in late passage normal human primary fibroblast cells. (**A**) Analysis of indirect immunofluorescence with anti-TRF2-2meR17 antibody in normal human primary fibroblast AG02261 cells at either p15 or p45. Cell nuclei were stained with DAPI in blue. (**B**) Senescence-associated β-galactosidase assays for AG02261 cells at either p15 or p45. (**C**) Quantification of percentage of AG02261 cells with altered nuclear staining of methylated TRF2. A total of at least 900 cells in triplicate were scored in blind for each passage as indicated. Standard deviations from three independent experiments are indicated. (**D**) Quantification of percentage of normal human primary fibroblast GM1706 cells with altered nuclear staining of methylated TRF2. A total of at least 900 cells in triplicate were scored in blind for each passage as indicated. Standard deviations from three independent experiments are indicated.
